# Biosorption characteristics of a highly Mn(II)-resistant *Ralstonia pickettii* strain isolated from Mn ore

**DOI:** 10.1371/journal.pone.0203285

**Published:** 2018-08-31

**Authors:** Huimin Huang, Yunlin Zhao, Zhenggang Xu, Yi Ding, Wan Zhang, Liang Wu

**Affiliations:** 1 College of Bioscience and Biotechnology, Hunan Agricultural University, Changsha, China; 2 Hunan Research Center of Engineering Technology for Utilization of Environmental and Resources Plant, Central South University of Forestry and Technology, Changsha, Hunan, China; 3 School of Material and Chemical Engineering, Hunan City University, Yiyang, Hunan, China; Fred Hutchinson Cancer Research Center, UNITED STATES

## Abstract

Microorganisms play an important role in immobilizing and detoxifying excessive Mn; however, there is so far a lack of sufficient information concerning highly Mn(II)-tolerant bacteria. The present study was conducted to analyze the bio-sorption characteristics of a strain (HM8) isolated from manganese ore wastes. Analytical data from the 16S rDNA sequence determination showed that the species, HM8, had a 99% similarity to *Ralstonia pickettii*. Results from the designed physiological, biochemical and isothermal adsorption tests indicated that HM8 did not only grow well at a Mn(II) concentration level of 10,000 mg/L but also removed 1,002.83 mg/L of Mn(II) from the bulk solution of the culture, showing that the isolated strain possessed strong capabilities to tolerate and remove Mn(II). In the isothermal bio-sorption experiments performed to investigate the effects of relevant factors on Mn(II) sorption, the highest Mn(II) removal rate was obtained at the contact time 72 h, temperature 40°C, and pH 6.0, while the differences in both strain growth and Mn(II) removal rate between different inoculated HM8 doses were found to be insignificant within the tested range. Scanning electron microscopy showed that, under Mn(II) stress, HM8 cells appeared irregular and cracked, with apparent wrinkles on the surface. The peaks in the Fourier transform infrared spectra showed that hydroxyl and carboxyl groups were the main functional groups for Mn(II) adsorption. The experimental data supported the practical application of HM8 as a biological adsorbent for remediation of heavily Mn contaminated sites.

## 0 Introduction

Heavy metal pollution, especially the pollution from metal mining activities, is of a great concern in the world [1, 2]. Not only does the metal pollution affect the quality of the water bodies, atmosphere, and food crops but also threatens the health and well-being of animals and human beings [3, 4]. Many studies have shown that most of the heavy metal pollutions in the environment were caused by anthropogenic sources, especially mining and smelting operations of metal ores [5–7]. Rapid increase in heavy metal contaminated wastelands due to mining activities in different countries has become a severe worldwide problem [8]. In addition, large quantities of untreated wastewaters brought about from mining and smelting operations yield another major global problem that can result in potential hazard to the natural environment [9].

Manganese (Mn) has different oxidation states, which play important roles in maintaining the normal structure of plant chloroplast membrane. As a necessary element for plant growth, Mn directly participates in plant photosynthesis. As a micronutrient required in small quantities, Mn is also essential for both growth and survival of humans, fungi and bacteria. Excessive amount of Mn, however, makes the metal dangerous to human health, plants, and microorganisms. Therefore accumulation of Mn at toxic levels can result in destructive effects on ecosystems [10]. Normally, heavy metals in soil and water can hardly be removed and degraded. The accumulated metals in the contaminated sites may final enter the food chain and cause toxic, carcinogenic and mutagenic effects. Researches have shown that there is a positive correlation between metal environmental exposures and development of neurodegenerative diseases [11–13]. Exposure to Mn(II) has been involved in neuro degeneration associated with impaired dopaminergic, oxidative stress, mitochondrial dysfunction and neuro inflammation [14].

Face to heavy metal pollution problems, numbers of conventional methods have been reported either for removal or detoxification of Mn(II) from contaminated soils such as ion exchange, chemical precipitation, filtration, membrane separation, electro dialysis, evaporation recovery, reverse osmosis and chemical oxidation-reduction. However, most of the traditional methods mentioned above are found to be expensive and inefficient, and produce secondary pollutants such as toxic by-products [15, 16]. Researches have showed that large groups of microbial species, like bacteria, fungi and yeasts, possess tolerant and resistant natures to multiple heavy metals and have thus potential functions that can be applied for bioremediation of metal contaminated sites [16]. Soil microorganisms can affect the states and activities of heavy metals through various reactions including oxidation, reduction and bio-sorption and fixation. Biological absorption is one of the important pathways to immobilise heavy metals and reduce their toxicity. Soil amending using bio-sorbents can promote the growth of the plants and further enhance their ability to absorb and assimilate heavy metals in the soil [17–19]. In the early 1980s, some scholars proposed the use of microbes to control heavy metal-contaminated soil because of the ability of microorganisms to fix toxic heavy metal ions and convert them into non-toxic or low-toxic elements [20]. The absorption of heavy metals by microorganisms is mainly divided into two steps: the first step is not related to the metabolism but to the biological adsorption process [21], while the second step involves the biological accumulation. The reaction in the processes is slow and is considered the main way for microorganisms to absorb, transform, and use heavy metal ions [22]. The microbial accumulation process is directly related to cell metabolism, because the activities of the microorganisms require the participation of metal ions, and when the cells transport these metal ions, some metal ions compete for the adsorbed sites [23]. Therefore, there are many factors affecting the cell biological activity such as initial ion concentration, contact time, temperature, inoculation dose, and pH, while the factors that have significant impacts on the detoxification function of microorganisms need to be investigated [24].

The mining area is a valuable microbiological library, where microbial resources can not only be used as bioremediation tools, but also guarantee ecological security. Biological remediation of toxic metal-contaminated soil is a convenient method to treat heavy metal pollution as it is an inexpensive environmental friendly natural process and has high public acceptance [25]. In recent years, many researchers have conducted anti-manganese microbial screening and functional utilization work. Han et al. [26] isolated three strains with strong ability to remove Mn(II) from Mn(II) contaminated soil, which were identified, respectively, as Oxidizing *bacilli*, *Xylose colorless* and *Serratia marcescens*. El et al. [27] found that *Serratia sp*. MSMC541 was resistant to several heavy metal species (As, Cd, Cu, Pb, Zn) and the cell walls of the strain possessed high metal adsorption capacities. Hassimi [28] showed that there was a simultaneous biosorption of Pb and Mn by *Bacillus cereus* while its biosorption efficiency was subject to environmental factors. Li Huidong [29] identified a strain with the highest Mn(II) tolerance concentration of 80 mmol/L from Mn ore. A large number of heavy metal-tolerant bacteria have been tested at different concentration levels ranging from 200 mg/L to 4,000 mg/L. However, most of the tested metal concentrations were relatively low, within a variation range between 50–400 mg/L [30–36].

In order to obtain more detailed information on behaviours of Mn-tolerant microbial species under highly Mn contaminated conditions, the present study was conducted to analyse the bio-sorption characteristics of a bacterium strain isolated from manganese ore wastes. Relevant tests including 16S rDNA sequence analysis were performed to determine the physiological and biochemical properties of the isolated strain. Isothermal adsorption experiments using the strain as a bio-sorbent were carried out to determine its capabilities to tolerate and remove Mn(II) within a wide Mn(II) concentration range. Kinetic adsorption model was applied to analyze the mechanisms related to the metal removal in its bio-growth and sorption processes. Scanning electron microscopy (SEM) analysis was further performed to observe the changes on cell surfaces after biosorption. In addition, fourier transform infrared (FTIR) techniques were used to determine the key functional groups of the bacteria that were responsible for immobilizing and detoxifying the metal. The main objective of the study was to provide basic and useful data for application of the isolated strain as a bio-sorbent for remediation of heavy metal contaminated soils.

## 1 Materials and methods

### 1.1 Collection of samples

Soil samples were collected from Xiangtan Mn ore wasteland, Hunan, China (112°45'E~122°55'E, 27°53'N~28°03'N), where the Mn(II) concentration in soil was up to 20,041 mg/Kg [37]. Soil samples were taken from 0–10 cm slag from Mn(II) tailings, passed through a 100-mesh sieve after mixing evenly, loaded in 50 mL centrifugal tube, and stored at −80°C.

### 1.2 Isolation of bacteria

The isolation of bacteria was performed in enriched Beef-Protein medium containing beef extract 0.3%, peptone 1%, and NaCl 0.5%, pH 7.2–7.6 (plus agar 1.5% for solid medium). The fresh samples (ten grams of each sample and prepared in triplicates) were incubated in 100 mL sterile water in triangular bottles spinning 120 rpm at 30°C for 12 h. Dilutions were prepared, and 0.5 mL of each diluted solution was incubated in solid medium with 500 mg/L Mn(II) (MnSO_4_·H_2_O, Xilong Chemical Co., Ltd, China) at 30°C for 72 h in a biochemical incubator (SPX-250B, China). Isolated colonies were incubated with increasing concentrations of Mn(II) (500 mg/L, 1,000 mg/L, 1,500 mg/L, 2,000 mg/L, 3,000 mg/L, and 4,000 mg/L) in solid medium at 30°C for 72 h.

### 1.3 Identification of the selected strain

#### 1.3.1 Bacterial characterisation

A highly Mn(II)-tolerant strain (HM8) was selected and characterised morphologically and biochemically. Gram staining, methyl red test, indole production test, hydrogen sulphide test, catalase test, oxidase test, Voges Proskauer test, glucose test, and gelatine liquefaction test were performed according to the standard methods [38].

#### 1.3.2 16S rDNA-based identification

The 16S rDNA was amplified by polymerase chain reaction (PCR) using two universal bacterial 16S rDNA primers: 1492R and 27F (1492R 5' TAC GGY TAC CTT GTT ACG ACT T 3' and 27F 5' AGA GTT TGA TCM TGG CTC AG 3'). The PCR was set up as follows: 10× Taq Buffer 2 μL, 2 mM deoxynucleotide triphosphates (dNTPs) 2 μL, 25 mM MgSO_4_ 1.2 μL, Taq enzyme 1 μL, 10 pm Primer1 1 μL, 10 pm Primer2 1 μL, Plate 1 μL, PCR Enhancer 5 μL, H_2_O 5.8 μL for 20-μL total volume. PCR amplification conditions were as follows: 96°C for 3 min, 35 cycles at 96°C for 20 s, 60°C for 30 s, and 72°C for 40 s, and 72°C for 5 min.

PCR products were detected by electrophoresis on 1% Tris base, acetic acid, and ethylenediaminetetraacetic acid (TAE) agarose gel, stained with ethidium bromide, and visualized using a Bio-Rad UV transilluminator (UVPEC3, USA). Sequencing (Illumina Hiseq 2500, California, USA) was performed by Shanghai Majorbio Bio-pharm Technology Company, Shanghai, China. The 16S rDNA sequences were compared to known sequences available in the GenBank 16S ribosomal RNA sequence database(https://www.ncbi.nlm.nih.gov/). The nucleotide sequences of 16S rDNA were aligned, and a phylogenetic tree was constructed using Mega 7.0 software based on the neighbour-joining method [39]. Tree topology was evaluated by bootstrap analysis based on 1,000 replicates [40].

### 1.4 Biosorption effect of Mn(II) concentration, inoculation dose, contact time, temperature, and pH

The bacteria were cultured on a rotary shaker (ZHWY-211C, China) (120 rpm) in a 250-mL conical Erlenmeyer flask containing 100 mL of Beef-Protein medium with different initial Mn(II) concentrations (0, 200, 400, 600, 800, 1,000, 2,000, 3,000, 4,000, 5,000, 7,500, and 10,000 mg/L) at an inoculation of 1 mL. For a better comparison [41, 42], the colonies in 100 ml beef peptone liquid medium (at 30°C in a rotary shaking incubator) was found to contain approximately 1.0 ×10^13^ cells/L (OD_600_ approximately 1.0). Results from previous tests indicated insignificant differences between cfu count and OD_600_ analyses. Following the methods applied in literatures [16, 43, 44], OD_600_ was thus applied to record the growth of bacteria in the present study.

The biosorption effect of the bacterial inoculation dose was explored, and the experiment levels were designed as 0.1, 0.5, 1.0, 2.0, and 3.0 mL of inoculation dose (corresponding, respectively, to 0.1%, 0.5%, 1.0%, 2.0%, and 3.0% of the total medium volume) with an initial Mn(II) concentration of 1,000 mg/L. Other culture conditions were the same as mentioned above.

Contact time effect on biosorption was investigated collecting samples every 6 h from 0 to 120 h, and other factors were the same as the first one with initial Mn(II) concentration of 1,000 mg/L.

The effect of temperature on biosorption was studied at 5, 10, 15, 20, 25, 30, 35, and 40°C, and the other settings were alike with previous.

The effect of different pH (3.0, 4.0, 5.0, 6.0, 7.0, 8.0, and 9.0) was tested on the samples. The initial pH of each metal solution was adjusted to the required pH by using 1 mol/L NaOH or 1 mol/L HCl. The other culture conditions were same as former.

In order to measure the removal rate of Mn(II) and strain growth in residual solution samples were collected at specific time points and centrifuged at 5,000 rpm for 10 min (TD5A, China). The supernatant fractions were separated and analysed for residual Mn(II)concentration using a flame atomic absorption spectrophotometer (AA7000, Japan). The pH was measured using the Rex laboratory pH meter (PHS-3C, China), and the optical density for strain growth at 600 nm (OD_600_) was measured using an ultraviolet spectrophotometer (UV-2450, China). All the tests were performed in triplicate. Variance analysis and data processing of Mn(II) uptake capacity and removal rate were conducted using statistical procedure SPSS 20.0. The removal rate, the growth of the strain, pH, and their interactions were analyzed by one-way or two-way analysis of variance (ANOVA), Pearson analysis method was applied to determine the correlation coefficient (r) and the least significant difference (*p*) [45].

$$q_{e}=\frac{V(C_{i}-C_{e})}{M};$$

$$Q=\frac{\left(C_{i}-C_{e}\right)}{C_{i}}\times 100\%$$

where q_e_ is the Mn(II) uptake capacity (mg/L), Q is Mn(II) removal rate, V is the volume of the Mn(II) solution (mL), C_i_ is the initial concentration of Mn(II) in the solution (mg/L), C_e_ is the final concentration of Mn(II) in the solution (mg/L), and M is the dry weight of the biomass (L).

### 1.5 Scanning electron microscopy (SEM) analysis

SEM analysis by JSM-6380LV, Japan was performed using samples before and after bio-sorption tests cultivated for 72 h under the conditions of initial Mn(II) concentration 1,000 mg/L, temperature 30°C and pH 5.6–5.8. The analytical condition parameters were as follows: backscattered electron mode (BSE), magnification of 10,000×, electron beam voltage of 3.0 kV, work distance of 8.5 mm, and temperature of 20°C. The bacterial samples were examined after cell fixation and vacuum freeze drying.

### 1.6 Fourier transform infrared (FTIR) analysis

FTIR was used to identify the main chemical functional groups of the strain. The function of some chemical groups present in the biomass was also identified by FTIR spectroscopy (NICOLET 5700, USA) using samples before and after bio-sorption tests cultivated for 72 h under the conditions of initial Mn(II) concentration 1,000 mg/L, temperature 30°C and pH 5.6–5.8. Bacterial samples before and after bioaccumulation were harvested by centrifugation at 8,000 × *g* for 10 min and mixed with 2% KBr. The mixtures were compressed into translucent sample disks and fixed in the FTIR spectrometer for analysis.

### 1.7 Metal sorption model

The biological adsorption isotherm is characterized by some constant numerical representations of the surface properties and affinity of the organism, and can be used as a comparison in biosorptive capacity of the biomass for heavy metals [46]. The biosorption isotherms of Mn(II) are commonly investigated using the Langmuir and Freundlich isotherm models to describe biological adsorption. The difference between the initial metal ion concentration and final metal ion concentration indicates the metal bound to the biosorbent [47]. In addition, the establishment of Langmuir and Freundlich model referenced Fan et al. [32].

## 2 Result and discussion

### 2.1 Selection of the optimal Mn(II)-resistant strain

A total of 23 Mn(II)-tolerant bacterial strains (HM1–23) were isolated from Xiangtan Mn ore wasteland with 500 mg/L Mn(II). Detection by concentration gradient method identified the highest resistant strain (HM8), which tolerated the Mn(II) concentration of 10,000 mg/L. Compared to other bacterial strains currently reported, HM8 showed high Mn(II) resistance[30–36].

### 2.2 Characterisation and molecular identification of HM8

The nucleotide sequence of the 16S rDNA of HM8 was submitted to GenBank (GenBank accession number MF536805). HM8 was identified as a rod-shaped bacterium. Gram-staining, methyl red, indole production, hydrogen sulphide and gelatine tests were negative, while catalase, oxidase, Voges Proskauer, and glucose tests were positive (Table 1).

**Table 1:** Morphological and biochemical characteristics of strain HM8.

Tests employed	Characteristics observed
Accession number	MF536805
*Morphology*	
Gram reaction	-
Shape	Short rod
Pigments	-
*Biochemical reactions*	
Methyl red	-
Indole	-
Hydrogen sulfide	-
Catalase	+
Oxidase	+
Voges Proskauer	+
Glucose	+
Gelatin	-

“+”and “–” indicate positive and negative reactions, respectively

**Fig 1:**
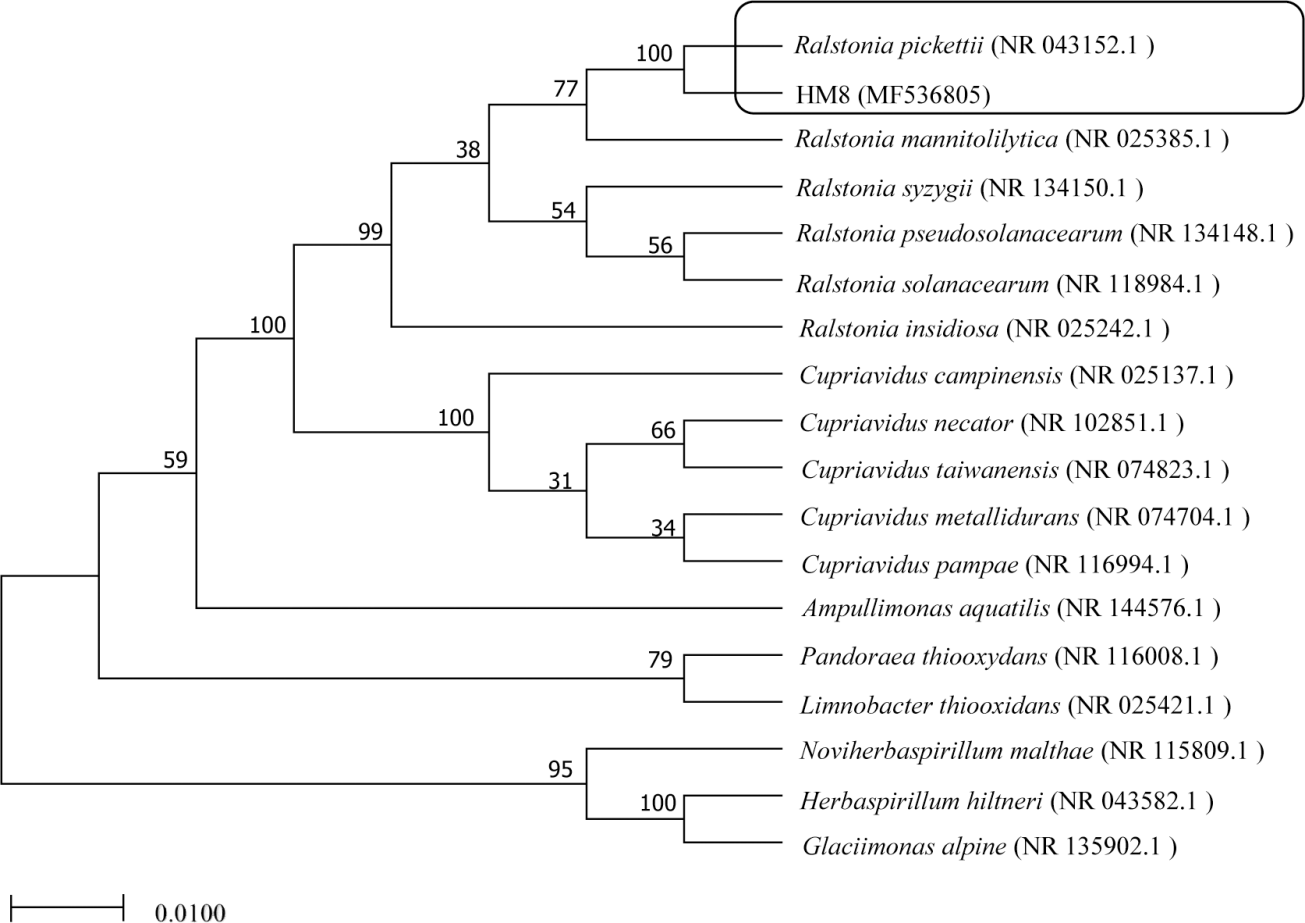
Phylogenetic relationships by a neighboring analysis of the 16S rRNA sequences showing the position of the strain HM8.

HM8 was subjected to 16S rDNA gene sequence analysis. Sequence analysis of the 16S rDNA is a fast and accurate method to identify the phylogenic position of bacteria. The partial 16S rDNA sequence of HM8 was uploaded to the National Center for Biotechnology Information (NCBI) website to search for similarity with known DNA sequences and to confirm the identity of HM8. The basic local alignment search tool (BLAST, https://blast.ncbi.nlm.nih.gov/Blast.cgi) search revealed that HM8 was closely related to *Ralstonia*, and it had 99% homology to *R*. *pickettii* (NR 043152.1). *R*. *pickettii* is able to live in areas with a very low concentration of nutrients, and several strains have shown an ability to survive in environments highly contaminated with metals [48, 49]. A phylogenetic (neighbour-joining) tree was constructed (Fig 1).

### 2.3 Characteristics of HM8 survival and biosorption under different initial Mn(II) concentrations

**Fig 2:**
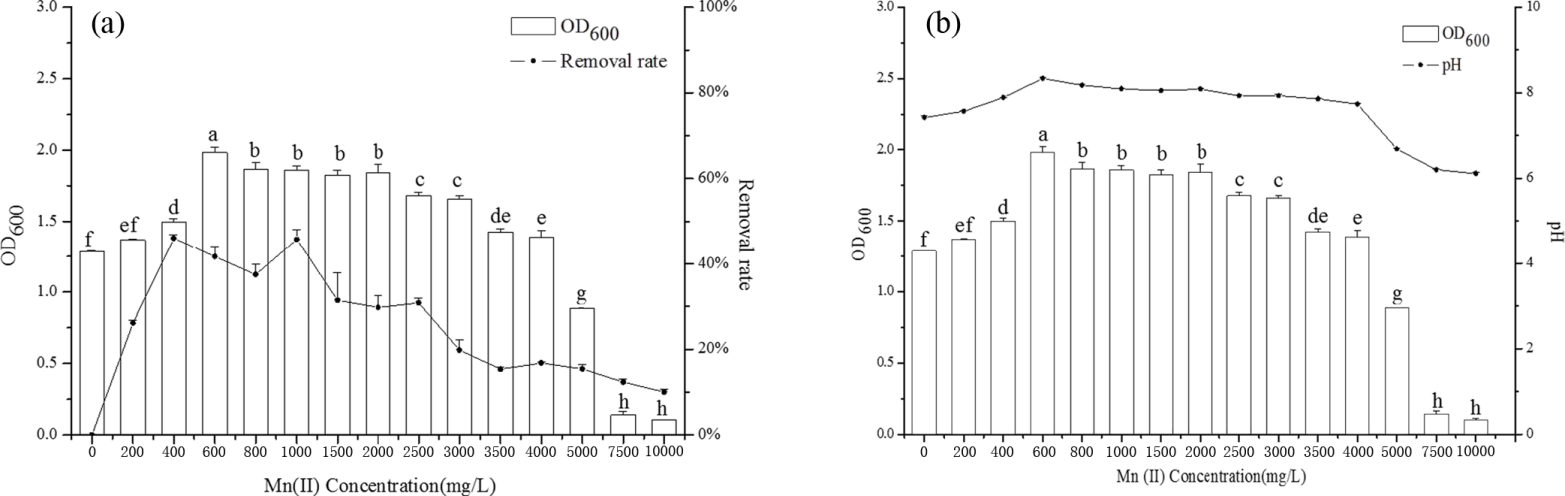
The relationship between HM8 growth, removal rate of Mn(II) and system pH at different Mn(II) concentrations.

The effect of the initial Mn(II) concentration on Mn(II) oxidation and reduction by HM8 was studied over a range of concentrations (0–10,000 mg/L). With the increase in Mn(II) concentration, the removal rate of Mn(II) firstly increased from 26.01% to 46.02% and then decreased to 10.03%, although it fluctuated at the concentration of 1,000, 2,500, and 4,000 mg/L. The best Mn(II) removal rate was up to 46.02% at a Mn(II) concentration of 400 mg/L (Fig 2A). On the other hand, the removal amount increased gradually and finally remained stable. At the highest Mn(II) concentration of 10,000 mg/L, HM8 still grew, and the Mn(II) removal amount was 1,002.83 mg/L. The strain with strong reaction on to the highly resistance of Mn(II) was rare in the current study. This was due to the interference between binding sites and inoculation dose or insufficiency of metal ions in solution with respect to available binding sites. According to Fan at al [32], these results indicated that the higher adsorption capacities can be obtained at higher initial Mn(II) concentrations, possibly due to the initial Mn(II) concentration provided a driving force for overcoming the mass transfer resistance between biosorption and biosorbent medium. The bacterial density (OD_600_) showed an upward trend within the tested Mn(II) concentration range 0–600 mg/L. As a material source of bacterial growth, Mn(II) could promote the growth of bacteria under the condition of low ionic concentration (0–4,000 mg/L). When the concentration was greater than 5,000 mg/L, there was a downward trend of OD_600_, indicating a growth inhibition effect. Therefore, HM8 showed having a strong ability to absorb Mn(II), and can uptake Mn(II) for self-metabolism. The above process could be considered to be one of the main mechanisms for the strain to remove Mn(II) from the liquid medium. High levels of metal ion absorptions by different microbial species were also observed in other studies [47, 50].

From the experimental results, growth of HM8 (OD_600_) correlated with pH, and HM8 may release alkaline substances during the growth (Fig 2B). At Mn(II) concentrations of 0–600 mg/L, OD_600_ and pH increased, while, at concentrations of 600–10,000 mg/L, OD_600_ and pH decreased, but still higher than the initial pH (medium with no bacterium inoculation). The initial pH of the medium was found to be 5.6–5.8, and measurements from control tests (medium with no bacterium inoculation) showed that the addition of Mn(II) (MnSO_4_) did not result in significant influence on medium pH. In the bio-sorption tests, there was a apparently positive correlation between medium pH and OD_600_ (r = 0.981, *p < 0*.*01*). In general, the observed trend was that the growth of the introduced bacterium enhanced the medium pH while the increment in pH decreased with increasing medium Mn(II) concentration. When the Mn(II) concentration in the culture was raised to 5,000 mg/L and above, there was a decline trend of OD_600_, which could be well attributed to the inhibition effect of high metal concentration on bacterium growth. This may be explained by the fact that the pH decreased sharply with the increase of Mn(II) concentration, and more hydroxyl ionized H^+^ were exchanged with Mn(II). Oxygen atoms in O–H can participate in the oxidation reaction of MnO_2_ ionic oxidation, resulting in low pH of the system [42]. Silva et al. [51], Khalilnezhad et al. [52] and Hasan et al. [53] also observed influences of bio-growth on pH in their cultivated fungi and bacteria media. Although concrete mechanisms need to be further investigated, the presence of functional groups such as amines, amides and carboxylic substances in the cell walls could react with the metal ions and lead to changes in solution pH. The obtained results were in consistency with the observations in SEM and FTIR analysis.

### 2.4 Characteristics of biosorption under different culture conditions

**Fig 3:**
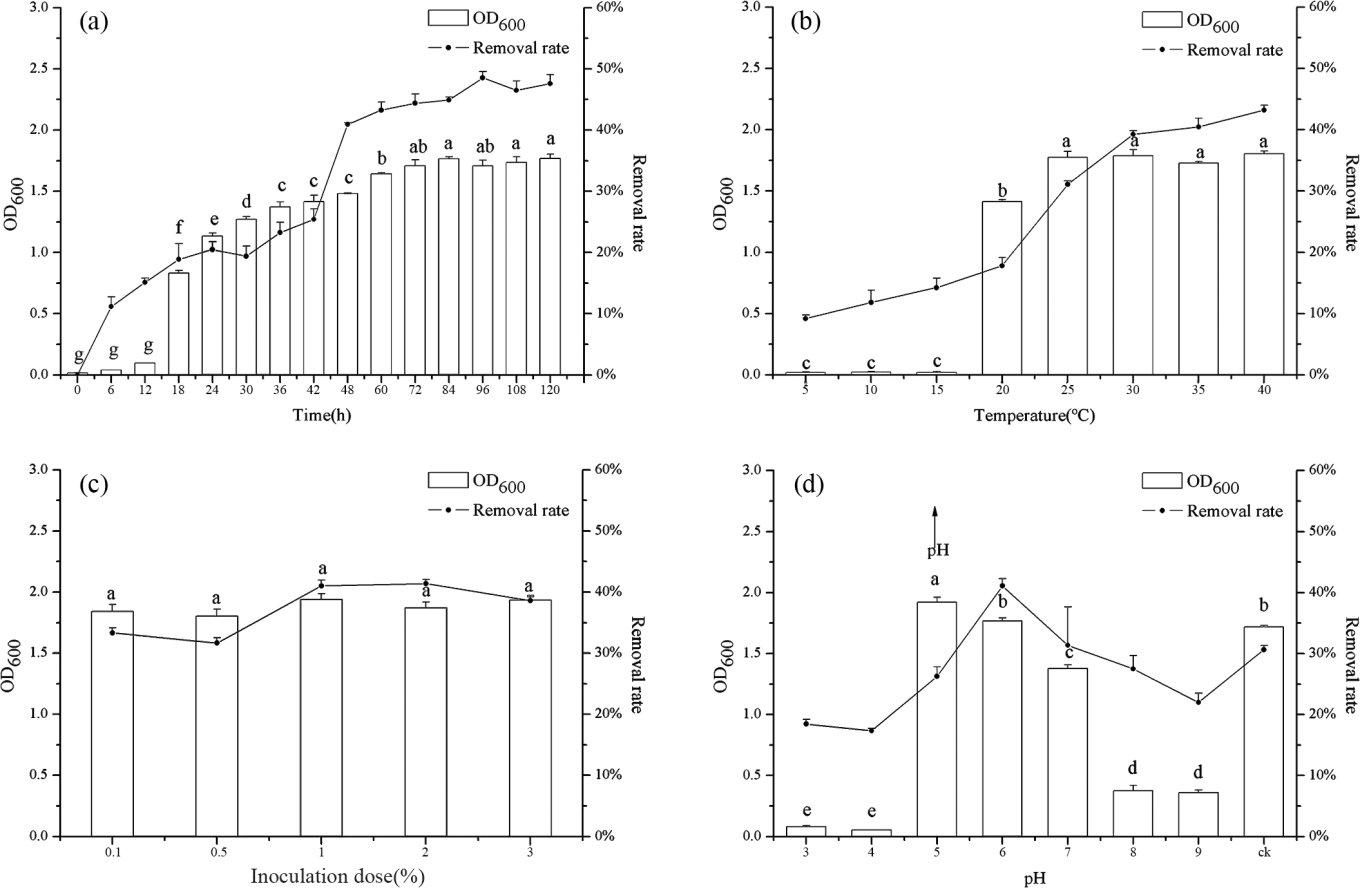
Single factor Experiment: (a) the relationship between HM8 growth and removal rate of Mn(II) at different time; (b) the relationship between HM8 growth and removal rate of Mn(II) at different temperature; (c) the relationship between HM8 growth and removal rate of Mn(II) at different inoculation dose; (d) the relationship between HM8 growth, removal rate of Mn(II) at different pH.

Contact time is one of the important factors of biosorption process [42]. The microbial growth and biosorption capacity increased with increasing contact times, the growth of HM8 (OD_600_) reached a stable phase in 72 h (Fig 3A), and the highest removal rate of Mn(II) was 48.54% in 96 h (the largest amount of Mn(II) was 485.36 mg/L).There was a positive correlation between the growth of HM8 and the removal of Mn(II) (r = 0.88, *p* < 0.01), indicating that the more biomass was present, the more the adsorption sites were, and the greater the removal rate was. After the equilibrium time was reached, no additional Mn(II) was adsorbed, indicating that the equilibrium Mn(II) concentration was presumably attained. In addition, HM8 growth was correlated to the pH (S2A Fig), suggesting that when the bacteria adsorbed Mn(II), some alkaline substances were released. Hence, the rate of Mn(II) biosorption was pretty high at the beginning time due to the high affinity of free Mn(II) binding sites on biosorbent, but after approximately 72 h the rate of biosorption slowed down and reached the equilibrium. In addition, Volesky [54] and Zoubolis [55] also indicated that the initial shortest time period of sorption process with the Mn(II) binding sites is significant for a high rate of metal sorption.

The effect of temperature on Mn(II) oxidation and reduction in the enrichment culture medium is shown in Fig 3B. At low temperatures (5–15°C), HM8 did not grow, and the removal rate and uptake amount of Mn(II) were extremely low. The removal rate of Mn(II) and the growth of HM8 increased with increasing temperatures from 20 to 40°C. The highest removal rate reached 43.20% at 40°C (432.02 mg/L removal amount of Mn(II)), and the growth of HM8 (OD_600_) reached a stable phase from 25 to 40°C. Besides, the growth of HM8 may correlate with the pH and release of alkaline substances (S2B Fig). So, the sorption of Mn(II) involved not only physical but also chemical sorption, the biosorption of Mn(II) was endothermic and some alkaline substances were released from HM8. Extreme temperatures totally reduce bacterial growth and Mn(II) oxidation due to the loss of viability or metabolic activity of cells during prolonged incubation. Compared with other studies, at low temperatures, the fluidity of the membranes weakens, preventing the function of the transport systems so that the substrates cannot enter into the cell rapidly and causes the low rate of growth [42, 44]. At temperatures higher than the optimum, it will lose the function of Mn(II) oxidation, alteration of membrane structure, or inactivation of protein synthesis due to the alteration of ribosome conformation occur [56].

The removal rate, final biomass, and pH were stable after 3 days of culture with different inoculation dose (Fig 3C and S2C Fig). With the increase of inoculation amount, the removal of Mn(II) by HM8 increased slightly, but the removal rate did not increase with the increase of the inoculation dose of the bacteria. When the amount of inoculation increased from 0.1 mL to 1 mL, the highest removal rate of Mn(II) was 41.01% (removal amount reached 410.08 mg/L), slightly higher than the others. There was no different interference between available binding sites and different inoculation dose or insufficiency of metal ions in solution. Based on the research from Fan et al. [32], this indicated that in the adsorption system determined by the concentration of the metal, as long as the surface adsorbed sites of the bacteria were not saturated. In the case of certain nutrients, the amount of bacteria increased the effect of the removal amount was not obvious. Therefore, in order to ensure a high removal amount and to save costs, the inoculum should not be too heavy.

Solution initial pH is a critical parameter for adsorption experiments. pH can affect the permeability of cell membranes, activity of functional groups, dissolution of intracellular substances, or ionisation [57]. For different strains, the optimum pH range is different, and understanding the suitable pH range of the strains is helpful for the application of microorganisms in industry. In this study, the pH had a great effect on HM8 adsorption ability (Fig 3D). When the pH was 6.0, the maximum removal rate was achieved (41.08%), with the removal amount of 410.817 mg/L. At the pH range 3.0–6.0, the rate of removal gradually increased, while at the pH range 6.0–9.0, the rate of removal decreased. In particular, with the initial pH of 5.0, HM8 reached the maximum growth amount, and the solution was alkaline (pH 7.81) (S2D Fig). So, HM8 grew better under weak acid conditions, and, after the reaction, the solution became alkaline, indicating that the bacteria may secrete alkaline substances. At low pH (< 5.0), the biosorption capacity for all metal ions is very low, because a large quantity of H^+^ competes with metal ions at sorption sites. As the pH increases, more negatively charged cells become available, facilitating a greater metal uptake. On the contrary, at higher pH values (> 7.0), more ligands like carboxyl, phosphate, imidazole, and amino groups are exposed and carry negative charges with a subsequent attraction of metal ions with positive charge, biosorption onto the cell surface, and metal precipitation at high pH values, inhibiting the contact of the metal with the biomass. Similar results have also been detected by Pardo et al. [58], and several published finding claim that pH plays an important role in affacting Mn(II) oxidation [51, 59, 60].

### 2.5 SEM analysis

**Fig 4:**
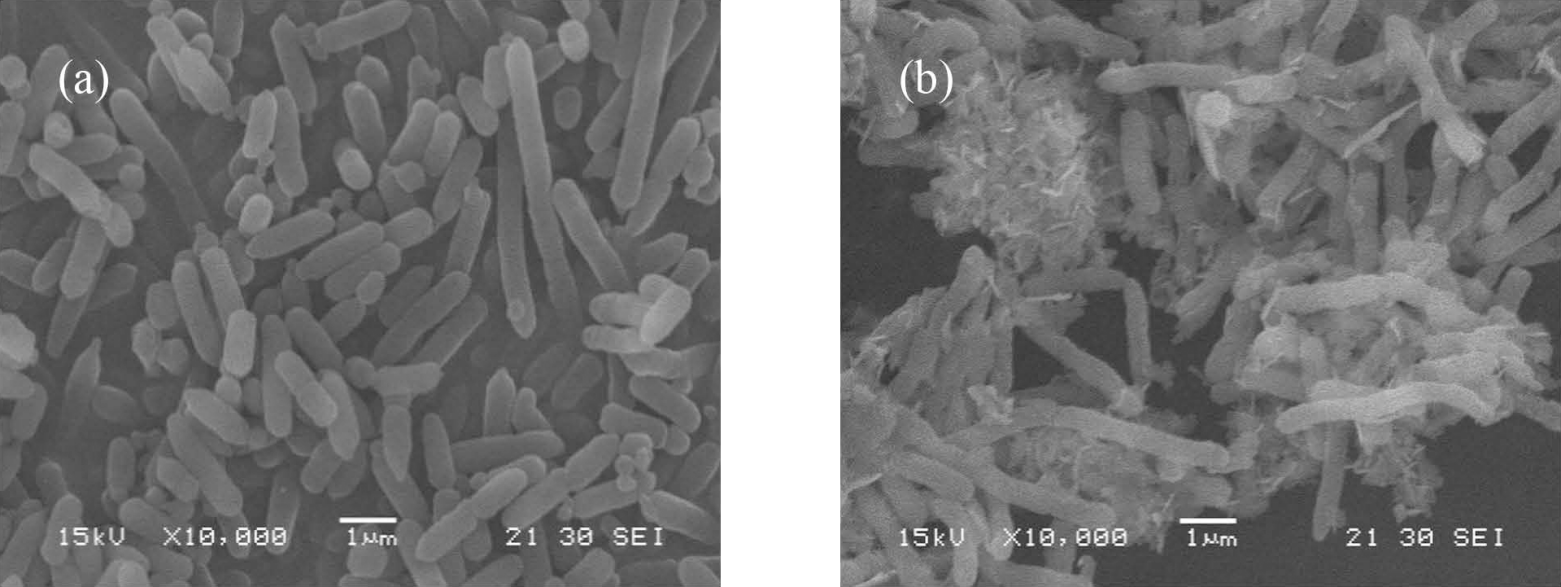
SEM microgragh of HM8: (a) before Mn(II) before biosorption; (b) after Mn(II) biosorption.

HM8 grown without Mn(II) and exposed to 1,000 mg/L Mn(II) was analysed using SEM at a magnification of 10,000×. The analysis showed that, without Mn(II), HM8 was morphologically rod shaped (Fig 4A), with a smooth surface, and the diameter of the cells was approximately 0.5 μm. The cell-surface morphology considerably changed after metal biosorption. The surface of metal-loaded cells looked vague and distorted, and seemed to be damaged by the heavy-metal ions. HM8 cells were irregular and cracked, with wrinkles on the surface after Mn(II) stress (Fig 4B). The small size of bacteria provides a large contact interface, which facilitates the interaction with metals for biosorption process. The alteration in morphology results from the secretion of extracellular polymeric substances during metal biosorption. In agreement to our findings, numerous researchers have showed the similar results [61].

### 2.6 FTIR analysis

**Fig 5:**
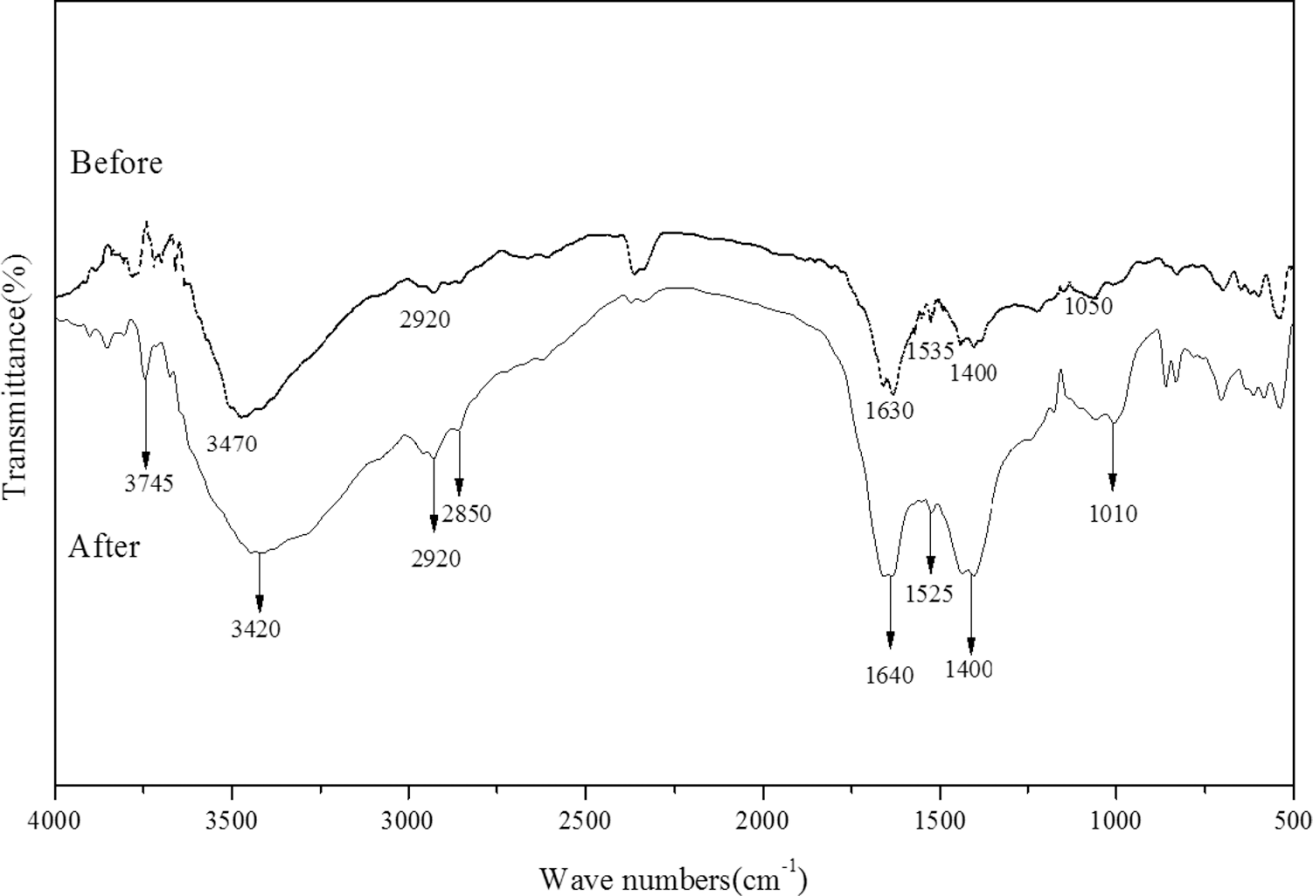
FTIR spectrum of HM8 before and after adsorption of Mn(II).

FTIR is an important tool for identifying functional groups of organic materials [62]. Visible changes in peak position and intensity were observed before and after adsorption of Mn(II) (Fig 5). The broad and intense adsorption peaks at 3400–3750 cm^−1^ corresponded to hydroxyl (OH–) stretching vibrations due to stretching [62]. Alkyl (C_n_H_2n+1_–) and aldehyde (–CHO) had a broad band ranging between 2,920 and 2,850 cm^−1^, resulting from proteins, carbohydrates, and other substances [63]. O = CN–H of amide groups showed peaks at 1,630 and 1,640 cm^−1^. The carboxyl (COO^-^) peak of the carboxylate groups appeared at 1,535 cm^−1^. Protein amide I band was present at 1,400 cm^−1^. Adsorption peaks at 1,000–1,200 cm^−1^ were the peak ranges of sugars [64]. The results indicated that the main constituents of HM8 and bacterial secretion were polysaccharide compounds containing a large number of hydroxyl groups contained in the pyrrole ring and proteins.

Comparison of FTIR spectra of HM8 before and after adsorption showed that, after adsorption of heavy metals, the peak shape was almost the same. After Mn(II) adsorption, the wavenumber of the hydroxyl group (blue) shifted from 3,470 to 3,420 cm^−1^ compared to the HM8 before Mn(II) binding, and 4,000 the peak intensity near 3,400 cm^−1^ decreased to some extent after Mn(II) binding. These results indicated the possible binding of Mn(II) to hydroxyl groups. The absorption peaks at 1,400 cm^−1^ of COO^-^ increased after Mn(II) absorption, suggesting that carboxyl groups may be involved in Mn(II) binding. In addition, there was no considerable change in the peaks of the amide bands after adsorption of heavy metals, indicating that proteins had little effect on adsorption. The results indicated that hydroxyl and carboxyl groups were the main active groups for adsorption or complexation of Mn(II). Figueira [65] pointed out that metal-binding is likely to form structures with carboxyl groups on bacterial cells, and other similar results have also been reported [16, 44, 53].

### 2.7 Biosorption isotherms

**Fig 6:**
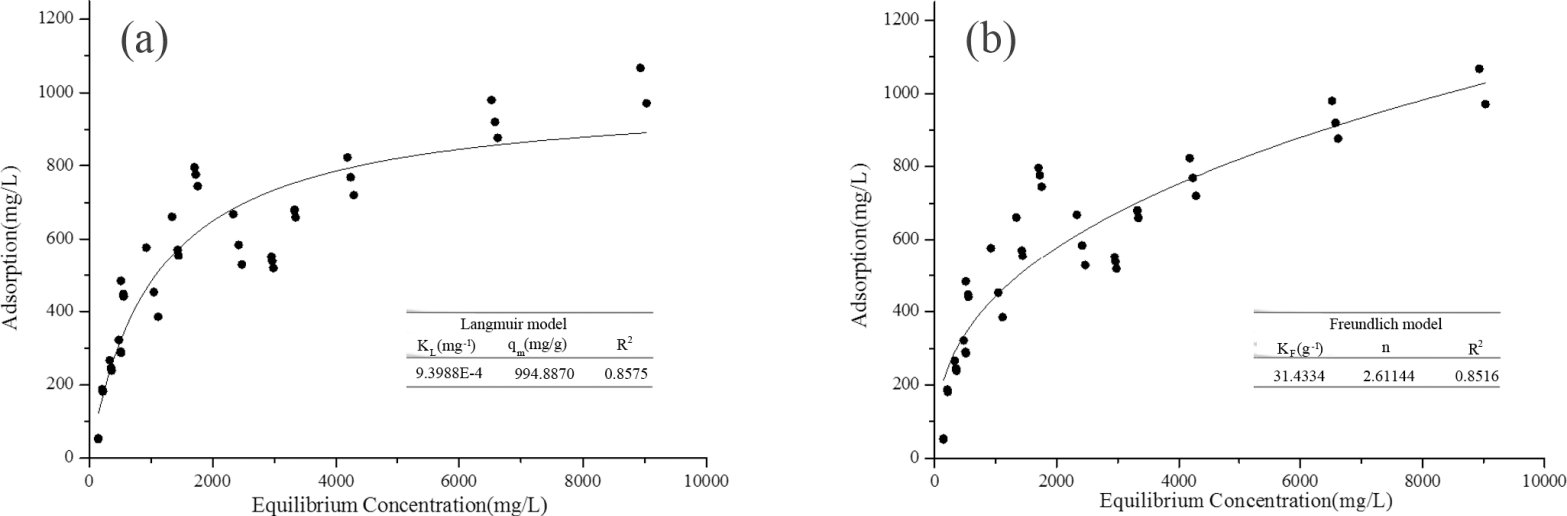
Isotherms of Mn(II) biosorption by Ralstonia pickettii HM8: (a) simulation with Langmuir isotherm model; (b) simulation with Freundlich isotherm model (symbols: experimental data; lines: model prediction).

A successful biosorption process can be better understand through the study of biosorption isotherms [66]. In this study, Langmuir and Freundlich isotherms were used to simulate Mn(II) adsorption process and linear fit of HM8 (Fig 6A and 6B). From simulation with Langmuir isotherm, the predicted maximum adsorption capacity (q_max_) was 994.8870 mg/L and was consistent with the actual situation. Comparing the two isotherms, the linear coefficient of determination (R^2^) of Langmuir (0.8575) was close to that of Freundlich (0.8516), both models showed a better absorption process as indicated by R^2^. According to Wang et al. [67], these results indicated that, with a decrease in metal ion concentration, the biosorption rate increased rapidly, while, with higher metal ion concentrations, a substantial decline in metal removal rate occurred until the equilibrium was reached probably due to the saturation of a number of adsorption sites. Biosorption of metals is better at low concentrations, probably because of the rapid metal absorption capacity of bacterial biomass. On the contrary, metal ions diffuse through the particles to the surface of the biomass at higher metal concentrations, and the hydrolysed ions slowly diffuse, resulting in a decrease in the adsorption rate. This phenomenon is generally related to the chemical mechanism of metal ions on the cell surface [68]. Furthermore, in the Langmuir model, K_L_ is the Langmuir constant related to adsorption capacity. When the value of K_L_ is high, the affinity of the metal ion for the biosorbent increases. In the Freundlich model, K_F_ indicates the degree of adsorption. When the K_F_ value is low, it means that the adsorption amount of heavy metals is small, and the higher the K_F_ value, the stronger the adsorption capacity [44]. These parameters can be used to compare the adsorption capacity of HM8 with different species or between different heavy metals. Therefore, Langmuir and Freundlich may both satisfy the adsorption process, the absorption of Mn(II) mainly effected by single layer adsorption of HM8, and more adsorption mechanisms need further study.

## 3 Conclusions

As an alternative to basic chemical methods, bioremediation has proved to be a viable strategy for Mn (II) removal. Researchers have found that the widely occurring Mn oxides in the natural environment are mainly formed through the action of microorganisms, microorganisms can catalyze and oxidize Mn(II) to form oxides, which greatly increase the oxidation rate of Mn(II) [69, 70]. Further studies have found that the adsorption capacity of Mn oxides produced by microbial transformation is generally higher than that of chemically synthesized Mn oxides [71, 72], and the primary product of bio-manganese oxidation has the activity of catalytic oxidation of Mn(II) [73]. Therefore, the characteristics and applications of microbial removal of Mn have gradually become the current research hotspot. In this research, it has been found that the *R*. *pickettii* HM8, a high Mn(II)-resistant strain isolated from Mn ore, was able not only to survive under a Mn(II) concentration of 10,000 mg/L but also to successfully remove high amounts of Mn(II) from the tested aqueous solutions. Research shows that *R*. *pickettii* is an oligotrophic organism that can survive in environments with low nutrient concentrations. Some strains have been proved the ability to persist in high concentrations of metal-contaminated conditions, the capability to survive in the harsh environments makes *R*. *pickettii* a candidate for bioremediation [74–76]. In comparison with other species reported in literatures [30–36], HM8 was shown to possess much stronger capabilities to tolerate and remove Mn(II) under heavily Mn polluted conditions. In the present study, the results indicated that the Mn(II)-resistant strain HM8 was closely related to *R*. *pickettii*. It survived under an extremely high concentration (10,000 mg/L) of Mn(II) and successfully removed Mn(II) at a rate of 1,002.83 mg/L. This situation was rarely seen in the current study and far exceeded the tolerance of ordinary bacteria to Mn(II). At the concentration of 1,000 mg/L of Mn(II), HM8 entered a stable phase in 72 h, and the removal rate of Mn(II) increased with increasing temperature from 20 to 40°C and 40°C up to the maximum rate of removal. HM8 grew better under weak acid conditions, and the highest rate of removal was at pH 6.0. Besides, with the initial pH of 5.0, the solution pH considerably increased (pH 7.81) after reaction, indicating that the bacterial growth may secrete alkaline substances. While the differences in both strain growth and Mn(II) removal rate between different inoculated HM8 doses were found to be insignificant within the tested range. The adsorption isotherms indicated that the absorption of Mn(II) mainly effected by single layer adsorption at varying Mn(II) concentrations. SEM micrographs showed that HM8 cells were irregular and cracked, with wrinkles on the surface after Mn(II) stress. The functional groups identified on the bacterial surface by FTIR included hydroxyl and carbonyl groups, which may be involved in the biosorption of Mn(II). Mn(II) could promote the growth of the strain under the condition of a certain ionic concentration (0–4,000 mg/L). With the removal of Mn(II), the strain produced a precipitate, and the pH of the solution gradually increased with the growth of the strain. It can be inferred that the Mn(II) removal mechanism of this strain is related to the biosorption, bio-enrichmentn and oxidation of the microorganism itself [77]. The results showed that HM8 can be used as a cheap and reliable biological adsorbent in soil Mn ore remediation. Compared to other studies, the results obtained from this study are promising, but the complete mechanism underlying Mn(II) reduction needs further investigation.

## Supporting information

S1 Fig Colony morphology of dominant strains. (TIF)

S2 Fig Single factor Experiment: (a)The relationship betwee*n* HM8 growth and pH at different time; (b)The relationship between HM8 growth and pH at different temperature; (c)The relationship between HM8 growth and pH at different inoculation dose; (d)The relationship between HM8 growth and pH at different pH.
(TIF)

S1 Table The experimental data of HM8 characteristic biosorption. (XLSX)
