# Supplementary figures and images for: Biosorption characteristics of a highly Mn(II)-resistant Ralstonia pickettii strain isolated from Mn ore
Source: PLoS One. 2018 Aug 31;13(8):e0203285. doi: 10.1371/journal.pone.0203285 (PMC6118360; doi:10.1371/journal.pone.0203285)

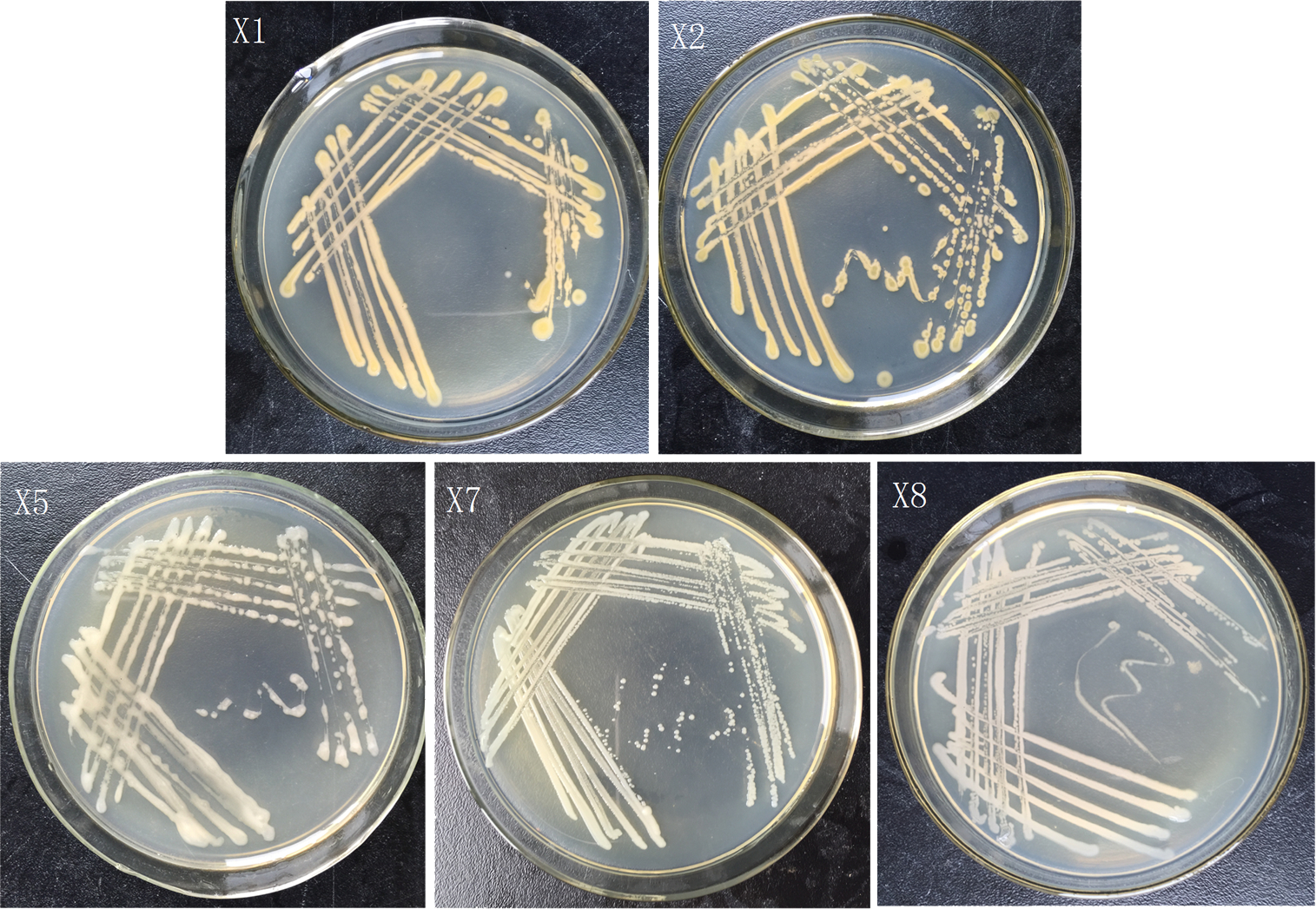

Supplement: S1 Fig — (TIF) [file pone.0203285.s001.tif]

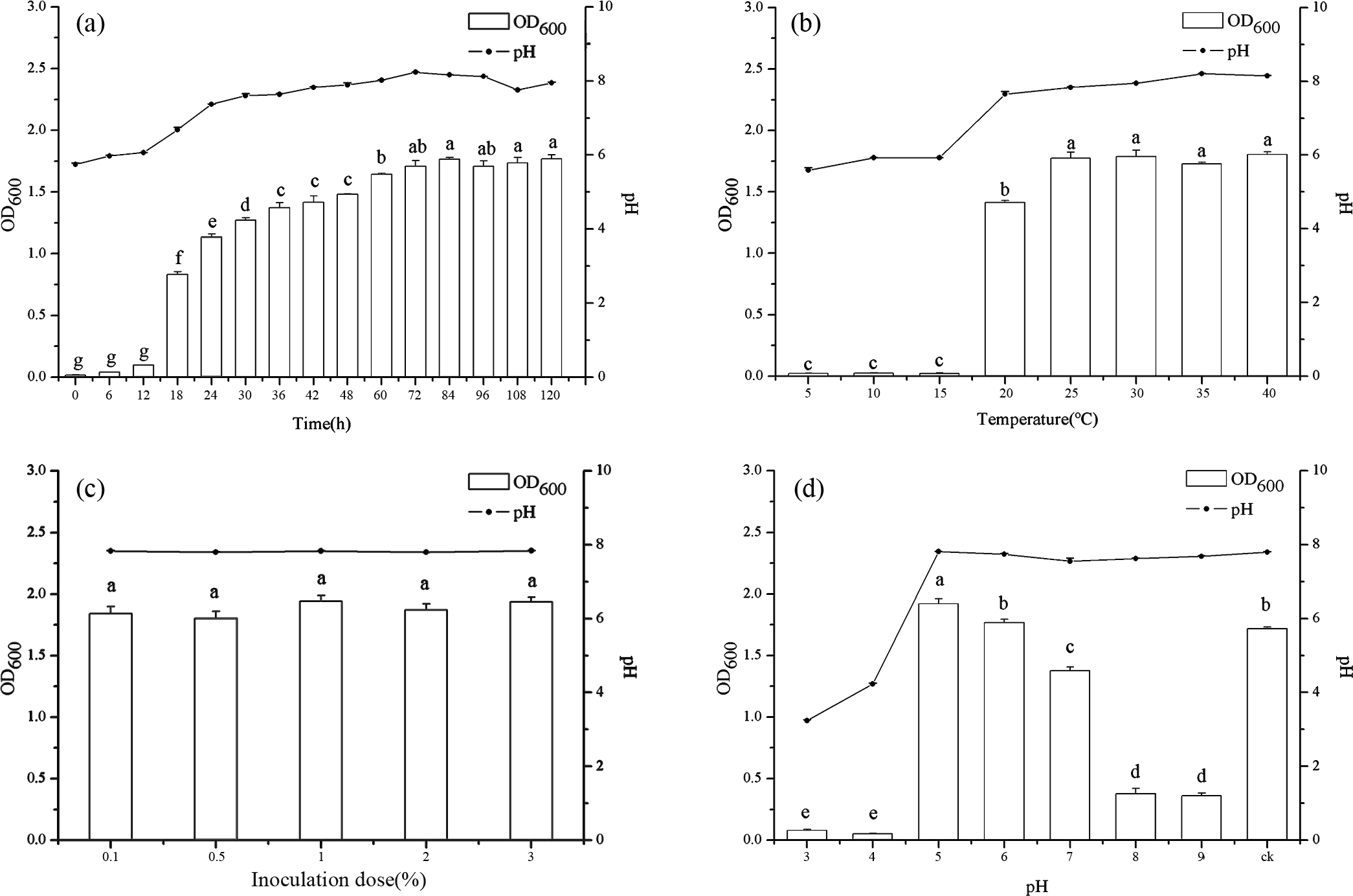

Supplement: S2 Fig — Single factor Experiment: (a)The relationship between HM8 growth and pH at different time; (b)The relationship between HM8 growth and pH at different temperature; (c)The relationship between HM8 growth and pH at different inoculation dose; (d)The relationship between HM8 growth and pH at different pH. (TIF) [file pone.0203285.s002.tif]
